# Predicting imminent suicide risk in a crisis hotline chat using machine learning

**DOI:** 10.1038/s41598-025-28704-0

**Published:** 2025-12-29

**Authors:** Yossi Levi-Belz, Meytal Grimland, Yael Segal-Elbak, Noam Munz, Hadas Yeshayahu, Joy Benatov, Avi Segal, Loona Ben Dayan, Inbar Shenfeld, Kobi Gal

**Affiliations:** 1 The Lior Tsfaty Center for Suicide and Mental Pain Studies, University of Haifa, Haifa, Israel; 2 School of Therapy, Counseling and Human Development, University of Haifa, Haifa, Israel; 3 The School of Behavioral Sciences, The Academic College of Tel Aviv–Yaffo, Yaffo, Israel; 4 Department of Software and Information Systems Engineering, Ben-Gurion University of the Negev, Beer-Sheva, Israel; 5 Department of Special Education, University of Haifa, Haifa, Israel; 6 SAHAR, Online Mental Support, Tel Aviv, Israel; 7 School of Informatics, University of Edinburgh, Edinburgh, UK

**Keywords:** Imminent suicide risk, Suicide ideation, Machine learning, Hotline, Human behaviour, Psychology, Risk factors, Signs and symptoms

## Abstract

Identifying individuals at the highest risk of suicide in real time is one of the critical tasks in suicide prevention. However, understanding the mental processes of at-risk individuals is a formidable endeavor due to the rapid, impulsive, and temporally dynamic characteristics of severe suicide crises. Recent theories, such as the *suicide crisis syndrome,* have suggested sets of short-term predictors of *imminent suicide risk* (IMSR), defined as an acute state that requires urgent intervention to prevent suicide. However, to date, no study has examined the prediction value of these theories in real time. In this study, we used machine learning to investigate its potential for predicting IMSR during internet-based crisis hotline chat sessions. We analyzed 3309 anonymized chat sessions from an internet-based crisis hotline, 312 of which were classified as IMSR chats (i.e., requiring immediate intervention). We compiled a lexicon of psychological factors derived from the main theories of suicide crisis and extracted language patterns associated with these key theoretical constructs. A logistic regression model within a machine learning framework was used to determine the odds ratio of each predictor, while temporal analysis was used to examine the stability of predictors throughout the chat duration. Suicidal ideation with a specific plan and intent emerged as the strongest predictor of IMSR. Additionally, pain tolerance and deliberate self-harm, components of acquired capability, were significantly associated with IMSR, aligning with the interpersonal theory of suicide. Cognitive rigidity and impulsivity, markers of cognitive deficiencies, also played a key predictive role. Conversely, perceived burdensomeness, depressive symptoms, and emotional pain were negatively associated with IMSR. Temporal analysis revealed that most factors remained stable throughout the chats. IMSR is best understood through an integrated approach that combines cognitive, affective, and behavioral components from multiple theoretical frameworks. As many individuals do not explicitly disclose their suicidal intentions, it is crucial to identify indirect risk factors to improve real-time risk detection. This study advances the theoretical understanding of imminent suicide risk and the development of practical tools for real-time crisis intervention through machine learning-driven analysis of crisis chat interactions.

**Supplementary Information:**

The online version contains supplementary material available at 10.1038/s41598-025-28704-0.

## Introduction

Suicide remains one of the most pressing global public health challenges, with approximately 700,000 lives lost annually^1^. The ability to accurately identify and respond to imminent suicide risk (IMSR)—defined as an acute risk of suicide occurring within hours or days—represents a critical frontier in suicide prevention efforts. Despite advances in suicide risk assessment, mental health professionals continue to face significant challenges in distinguishing between individuals at immediate risk of suicide attempt and those experiencing longer-term suicidal ideation^2,3^. This gap in clinical assessment capabilities has profound implications, as the hours and days surrounding acute suicidal crises represent crucial windows for life-saving interventions^4^. Therefore, understanding the unique predictors of imminent suicide risk is essential for developing more effective assessment tools and intervention strategies^5^.

Recent theories have focused on factors that may explain the specific mental state in which general suicide risk (GSR) transforms into an imminent one. Galynker et al.^6^ introduced the concept of the suicide crisis syndrome (SCS), referring to the acute mental state that precedes suicide attempts. SCS comprises five key components: pervasive feelings of frantic entrapment, affective disturbance, loss of cognitive control, hyperarousal, and social withdrawal. Recent reports have indicated that these factors can predict suicidal behavior in psychiatric inpatients during the 4–8 weeks after their hospital discharge [e.g. 6]. This predictive power is significantly greater than that of traditional personal history factors^7^. Although these reported findings are promising for assessing IMSR among psychiatric patients, predicting IMSR among the general population remains unclear.

Another relevant dimension is the *acquired capability*for suicide, highlighted by theories such as the interpersonal theory of suicide [e.g^8^. and the three-step theory^9^. Acquired capability is defined as an individual’s habituation to pain, fear, and death through exposure to life experiences such as non-suicidal self-injuries (NSSI). Empirical data have demonstrated that acquired capability can significantly differentiate suicide attempters from suicide ideators [e.g^10^].

In addition to these theoretical frameworks, validated assessment tools have been developed to systematically capture acute suicide risk markers. The Columbia-Suicide Severity Rating Scale [C-SSRS^11^;, widely regarded as the gold standard for suicide risk assessment in both research and clinical practice, operationalizes key dimensions such as suicidal intent and planning. Although not a theoretical model, the C-SSRS provides structured evidence that the presence of a suicide plan (i.e., detailed method and intended timing), especially when combined with a prior attempt, confers markedly elevated near-term risk requiring urgent intervention. The predictive utility of these C-SSRS dimensions has been demonstrated in large-scale clinical and epidemiological studies^11,12^, as well as in high-risk and emergency samples where ideation severity, intent, and behavior predicted imminent suicidal behavior^13–15^.

While these theoretical frameworks contribute to understanding what distinguishes individuals at imminent suicide risk from those at general suicide risk, accurately predicting suicide remains a weighty challenge^16–18^. In recent years, artificial intelligence (AI) and machine learning (ML) techniques have been increasingly utilized in mental health research to significantly improve the ability to detect and diagnose mental health conditions^19–21^. Large-scale studies have demonstrated their potential in adult populations, such as the Army STARRS project predicting suicide after psychiatric hospitalization^22^ and subsequent critical reviews emphasizing both promise and limitations^23^. As highlighted by Kirtley and colleagues^24^, ML applications in suicidology have expanded rapidly, showing good predictive accuracy yet limited clinical translation. Building on this foundation, more recent studies have leveraged diverse big-data sources, including national mortality records^25^, free-text crisis conversations^26^, and voice biomarkers^27^. Collectively, these findings demonstrate that ML can provide valuable assessments of an individual’s suicide risk, whether through structured administrative data, linguistic signals, or behavioral markers, thereby offering novel pathways for monitoring and intervention^27–29^.

One promising approach leverages language to gain insight into psychological experiences across various settings^30,31^. Advances in natural language processing (NLP) have enabled the development of algorithms that extract multiple parameters from the human language by introducing innovative methods. Notable advancements include a natural language processing AI model trained on patient portal messages, which predicted 30-day suicide-related events with accuracy comparable to that of widely used suicide assessment tools^32^. In another study, ML was used to analyze unstructured clinical notes from veterans’ medical records, achieving over 65% accuracy in predicting suicide risk and showcasing the potential of text analytics for clinical screening and monitoring^33^. Together, these findings highlight the growing role of ML and NLP in advancing suicide risk detection and prevention strategies.

One of the avenues for exploring NLP algorithms is crisis hotlines, which provide a real-time, text-based interaction environment. These features make them an invaluable source of naturalistic, high-risk language data that NLP models can analyze to identify linguistic markers of imminent suicide risk. Crisis hotlines carry considerable importance in the chain of care for IMSR individuals^34^, as they provide 24/7 access to para-professional support, guidance, and acute interventions^35^. Owing to the harsh and rapid nature of the suicide crisis, which often does not allow sufficient time to access professional help, the contribution of hotlines is critical.

### The current study

This study aimed to assess the relative contributions of various theory-driven factors in predicting imminent suicide risk (IMSR) by refining existing suicide risk theories and validating them in real-time crisis interactions. Specifically, we examined the extent to which factors from three major theories and frameworks that focus on the transition from ideation to action—the Suicide Crisis Syndrome (SCS), the Columbia framework of the Columbia-Suicide Severity Rating Scale (C-SSRS), and the Interpersonal Theory of Suicide—predict IMSR chats in crisis hotlines. For each of these theories and frameworks, we utilized a lexicon-based language analysis to represent their respective psychological constructs. Additionally, to broaden the scope of our investigation, we incorporated well-established predictors of general suicide risk (GSR), such as suicidal ideation and depression. Building on the results of this study, our aim was to develop a more comprehensive, evidence-based framework for assessing IMSR. Utilizing a unique dataset of chat sessions from an internet-based crisis hotline, this study bridges theoretical insights with real-world applications. We hope to enhance our understanding of the mental states preceding IMSR in acute settings.

## Methods

### Dataset

The study dataset is comprised of chat sessions of an internet-based crisis hotline chat service for distressed individuals (*Sahar*; https://sahar.org.il/about-us/). The website offers free online mental health support by text provided by a team of 400 qualified crisis volunteers. These crisis volunteers, trained and supervised by mental health professionals, provide anonymous support 24 h a day, seven days a week. Chat sessions were documented over a period of four years (January 2019 to August 2023). All chats were recorded and, as part of routine practice, manually labeled in real time by Sahar’s trained volunteers to capture the main presenting issues (e.g. suicidal ideation, domestic violence, marital conflict, anxiety, depression). The presence of a *suicidal ideation* label was used to classify chats as general suicide risk (GSR). These service-level labels were not used for the lexicon-based analyses presented in this paper. Volunteers complete a structured training program, including professional instruction, group simulations, supervised practice, and ongoing small-group supervision, to prepare them for crisis intervention work and to promote consistency in how presenting issues are identified and labeled. To validate the volunteers’ labeling, three clinical psychologists specializing in suicide prevention independently reviewed 600 randomly selected chat sessions (200 nonsuicidal, 200 general suicide risk [GSR], and 200 imminent suicide risk [IMSR]). They classified each chat into the same three categories (nonsuicidal, GSR, IMSR) without knowledge of the volunteers’ labels. Agreement between volunteer and expert classification was satisfactory (Cohen’s κ = 0.731), indicating adequate reliability of the service-level labeling. ome variation likely reflects the different contexts of labeling: volunteers coded chats in real time while engaged with the caller, whereas psychologists evaluated the transcripts retrospectively, without the immediacy of the live interaction. The average session length was 37 min. Upon entering the chat, the caller stated their age range and gender. There were 3309 chats overall: 2997 general suicide risk (GSR) chats and 312 imminent suicide risk (IMSR) chats.

### Outcome measure

An imminent suicide risk (IMSR) chat session was operationally defined as any chat session flagged as having a high probability of a suicide attempt or completion within a short timeframe ranging from hours to days. These chats were identified by volunteers in real time and reviewed in consultation with on-call supervisors, who are licensed mental health professionals. A mutual decision was made during the chat to escalate the case, leading to immediate involvement of the police or the online child protection bureau. For example, statements indicating imminent intent or behavior (e.g., “I am on my way to the bridge now” or “I just took 10 pills”) were characteristic of IMSR chats, whereas GSR chats typically involved suicidal ideation without urgent intent.

### Explanatory factors

The lexicon used for the chat analysis adopted language representations of prominent theories: the *interpersonal theory of suicide*[ITS; e.g^8^., *suicide crisis syndrome*[SCS^8^;, and the *Columbia-Suicide Severity*framework [C-SSRS^11^;, along with individual factors such as sexual harassment. Altogether, the lexicon comprises 18 key categories for IMSR. Moreover, we used 20 categories of factors which relate to GSR.

### IMSR categories

The ITS is represented by two components of fearlessness of death and pain tolerance, reflecting acquired capability (e.g., “Death doesn’t scare me”). The SCS is represented by the domains of affective disturbance, loss of cognitive control, disturbance in arousal, and social withdrawal. Each category includes its building blocks, such as agitation, hypervigilance, irritability, and global insomnia, which construct disturbance in arousal (e.g., “I feel so stirred up inside, I want to scream”). The C-SSRS includes active suicidal ideation with some intent to act without specific plan and active suicidal ideation with specific plan and intent (e.g. “working out the details of how to kill myself”).

### GSR categories

These included both theory-based constructs and empirically supported psychosocial risk factors. Two components of the ITS variables of thwarted belongingness and perceived burdensomeness (e.g., “These days I feel like a burden on the people in my life”), and suicidal ideation derived from the C-SSRS (e.g., “I have thoughts about killing myself”), were incorporated, together with psychosocial and clinical risk factors documented in prior research. These comprised past suicidal history, family suicide history, deliberate self-harm, loneliness, hopelessness, depressive symptoms, psychopathology, as well as empirically supported individual factors such as sexual harassment^36^, bullying and adverse life events^37,38^, LGBT-related stressors and immigration^39,40^, and impulsivity and perfectionism^41,42^. These key categories vary in length, with the shortest category including 20 phrases and the longest more than 400 phrases. For an extensive discussion of the psychological factor-based lexicon, please see our previous work^43^.

### Procedure

The lexicon was developed in three steps. First, language representations of the main psychological and empirically supported risk factors described above were generated. Second, we used validated questionnaires that tapped the theoretical constructs of the chosen main theories, such as the Acquired Capability for Suicide Scale [ACSS^44^;. Subsequently, 200 random chat sessions were scanned to identify more language representations reflective of theoretical factors. To further examine the reliability of the lexicon, two experts in suicidology independently coded 50 randomly selected chat sessions. Relevant sentences were labeled into the corresponding lexicon categories (e.g., “trying to relieve myself through cutting” coded as deliberate self-harm). Inter-rater reliability, calculated using Cohen’s kappa, was 0.811, indicating substantial agreement. In a complementary validation step, the same experts evaluated the representativeness of lexicon phrases across an additional set of 50 chat sessions. Each phrase was rated on a 1–5 scale for its relevance to the intended psychological construct, with an average rating of 4.14. This procedure supports both the reliability and the face validity of the lexicon categories. A detailed list of categories with representative phrases is provided in Appendix 1.

### Statistical analysis

We utilized a regression model within an ML framework to investigate the relative contribution of theory-driven factors to the model’s predictive performance. This analysis aimed to assess feature importance, providing insights into which predictors most significantly influenced the model’s ability to predict IMSR chats. Model performance was evaluated using receiver operating characteristic–area under the curve (ROC AUC), precision, and recall.

As a robustness check, we also applied regularization methods (lasso, ridge, elastic net). These did not improve classification performance, and lasso/elastic net produced unstable feature selection across splits, indicating limited added value in our data context.

Transcripts were analyzed automatically using a natural language processing (NLP) pipeline. A lexicon-based representation was generated for each chat, constructing a vector in which each entry reflected the frequency of expressions from a given lexicon category. The IMSR chats were oversampled to increase their representation due to the imbalance of the distribution. Specifically, each IMSR chat was sampled multiple times during training to ensure that the model was exposed to sufficient examples. This oversampling procedure helps prevent the model from being biased toward the more common non-IMSR chats and improves its ability to recognize rare but important cases. Oversampling the minority class is one of the most widely used techniques to address imbalanced datasets, particularly when predicting rare events such as IMSR chats^45^.

## Results

### Feature importance in predicting imminent suicide risk: logistic regression findings

The regression model demonstrated moderate performance in distinguishing IMSR from GSR chats, with a receiver operating characteristic–area under the curve (ROC AUC) of 68.8%. Precision was 46.7% and recall 49.2%. These performance metrics indicate that the predictive findings presented below should be interpreted with caution. Odds ratios were used as indicators of feature impact on model predictions, highlighting the relative contribution of each theory-driven feature to the model’s ability to classify chats. Table 1 presents significant predictors. The full list of predictors, including non-significant variables, is provided in Appendix 2. As can be seen on Table 1 language representations of *active suicidal ideation with a specific plan and intent* emerged as the strongest feature (OR = 1.767, 95% CI [1.6206, 1.9284]); *passive suicidal ideation* (OR = 1.301, 95% CI [1.1922, 1.4107]), was also a positive predictor of IMSR chats. *Pain tolerance*, a factor related to the acquired capability for suicide that predicts a higher risk for IMSR (OR = 1.212, 95% CI [1.0984, 1.337]). C*ognitive rigidity factor*, relating to the suicide crisis syndrome was the fourth significant predictor of IMSR chats (OR = 1.185, 95% CI [1.077, 1.302]).

**Table 1:** Logistic Regression Results: Key Predictors of Imminent Suicide Risk Chats (IMSR).

Feature language representations	Odds ratio	*p* Value	95% CI
Active suicidal ideation with specific plan and intent	1.767	0.00	(1.620, 1.928)
Suicidal ideation	1.301	0.00	(1.199, 1.410)
Pain tolerance	1.212	0.00	(1.098, 1.337)
Deliberate self-harm	1.143	0.00	(1.056, 1.237)
Cognitive rigidity	1.185	0.00	(1.077, 1.302)
Impulsivity	1.081	0.00	(1.003, 1.165)
Loneliness	1.159	0.04	(1.058, 1.269)
Perceived burdensomeness	0.881	0.03	(0.786, 0.985)
Emotional pain	0.872	0.01	(0.784, 0.970)
Irritability	0.815	0.00	(0.700, 0.946)
Sexual harassment	0.819	0.01	(0.732, 0.917)
Depressive symptoms	0.813	0.00	(0.736, 0.898)
Absenteeism	0.668	0.00	(0.570, 0.781)

95% CI = confidence interval, *p* < .05.

Other significant but somewhat weaker positive features were *loneliness* (OR = 1.159, 95% CI [1.058, 1.2697]), *deliberate self-harm* (OR = 1.143, 95% CI [1.0566, 1.2373]), and *impulsivity* (OR = 1.081, 95% CI [1.0032, 1.1654]). Conversely, some features exhibited lower odds ratios, reflecting their reduced likelihood of association with IMSR chats and, thus, were more likely to be associated with GSR chats. For example, *perceived burdensomeness* (OR = 0.881, 95% CI [0.7864, 0.9854]) is a theory-driven factor reflecting a component of the interpersonal theory of suicide. *Emotional pain* (OR = 0.872, 95% CI [0.7842, 0.9706]) is part of the affective disturbance of the suicide crisis syndrome theory, and *irritability* (OR = 0.815, 95% CI [0.7009, 0.9469]) that reflects the disturbance in the arousal component in that theory. Other features, such as *sexual harassment* (OR = 0.819, 95% CI [0.732, 0.917]) and *depressive symptoms* (OR = 0.813, 95% CI [0.7368, 0.898]), were also significantly associated with GSR chats. *Absenteeism* was the strongest negative predictor (OR = 0.668, 95% CI [0.5707, 0.7819]) of IMSR chats.

### Temporal dynamics of predictors for imsr chats: analysis across chat duration

We performed an additional ML analysis using logistic regression models to estimate the likelihood of IMSR at multiple time points throughout the chat. This approach allowed us to examine how the predictive power of each factor varied across the conversation, reflecting the dynamic unfolding of risk disclosures. Again, full results for all explanatory factors, including nonsignificant predictors, are provided in Appendix 2.

**Fig. 1:**
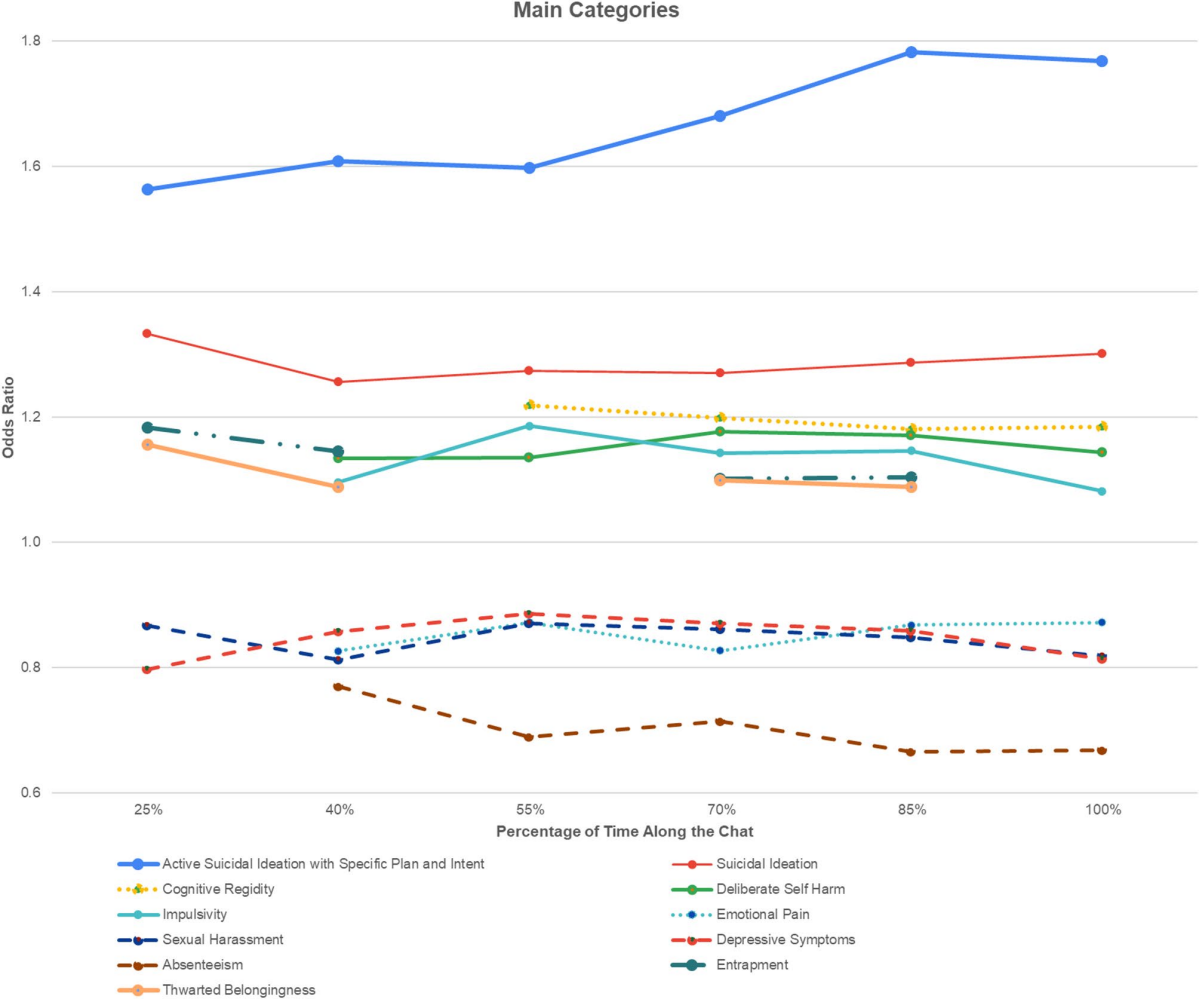
Temporal trends of significant predictors of imminent suicide risk (IMSR) over the course of the chat.

As shown in Fig. 1, the findings demonstrated consistency throughout the chats, as the predictors’ contributions were maintained from beginning to end. Thus, active *suicidal ideation with a specific plan and intent* feature (25%, OR = 1.563, 95% CI [1.4314, 1.7071]) and *suicide ideation* (25%, OR = 1.333, 95%CI [1.2342, 1.4398]) remained the strongest predictors of IMSR throughout the duration of the chat. The next strongest predictors were *cognitive rigidity* predicting IMSR mid chat (55%, OR = 1.218, 95% CI [1.078, 1.33]) and *entrapment* (70%, OR = 1.101, 95%CI [1.0134, 1.1974]).

Other significant factors were *deliberate self-harm* (40%, OR = 1.133, 95% CI [1.0496, 1.2248]), *impulsivity* (40%, OR = 1.095, 95% CI [1.0189, 1.1783]), and *thwarted belongingness* (40%, OR = 1.088, 95%CI [1.0027, 1.1814]), with the latter representing the interpersonal theory of suicide. *Emotional pain* (40%, OR = 0.826, 95% CI [0.7218, 0.9469]), a component of the suicide crisis syndrome, was found to predict non-IMSR chats by exhibiting a lower odds ratio halfway through the chat. Other theory-driven factors that showed a lower mid-chat odds ratio, indicating a lower probability of being associated with IMSR chats were *depressive symptoms* (40%, OR = 0.886, 95% CI [0.7814, 0.9428]), *sexual harassment* (40%, OR = 0.812, 95% CI [0.7149, 0.9242]), and *absenteeism* (40%, OR = 0.770, 95% CI [0.6606, 0.8978]).

## Discussion

This study aimed to advance the development of an empirically grounded model for predicting imminent suicide risk (IMSR) by leveraging real-time crisis hotline chat interactions. By applying machine learning (ML) techniques, we systematically analyzed linguistic representations and psychological constructs derived from established suicide risk theories and validated clinical frameworks. Using odds ratios, we quantified the relative impact of these theory-driven factors on IMSR prediction, providing a nuanced understanding of the cognitive, emotional, and behavioral indicators that may predict high-risk individuals in crisis settings.

Generally, the study’s findings validate the applicability of the major theoretical frameworks in understanding IMSR: the suicide crisis syndrome (SCS), the interpersonal theory of suicide, as well as the Columbia-Suicide Severity Rating Scale (C-SSRS), a widely used clinical assessment framework for evaluating suicidal intent and planning. *Active suicidal ideation with a concrete plan and intent* emerged as the strongest predictor of IMSR, reaffirming the central tenets of the Columbia framework^11^. This underscores the importance of structured risk assessment tools that prioritize suicidal intent and planning as critical risk factors^46^. At the same time, the strong association of this factor with IMSR likely reflects hotline procedures, in which such disclosures typically lead to escalation to the on-call clinical supervisor and, when imminent harm is suspected, referral to police. This procedural link may partly explain the predictive strength of the variable. More noteworthy, however, is that suicidal ideation without a plan was also associated with IMSR, despite not routinely triggering rescue protocols. This distinction highlights the central contribution of our study: identifying linguistic markers that differentiate general suicide risk (GSR) from imminent suicide risk (IMSR) beyond obvious procedural triggers.

In addition to explicit suicidal ideation, the findings highlight *pain tolerance* and *deliberate self-harm*—key elements of acquired capability for suicide—as significant predictors of IMSR. These findings align with the interpersonal theory of suicide^8^, which posits that individuals who develop a diminished fear of pain and death through repeated exposure to distressing experiences are at greater risk of enacting suicidal behaviors. It can be suggested that these findings have profound clinical implications, as individuals with a history of self-harm or high pain tolerance may bypass protective barriers, making early intervention critical^47^. Although acquired capability is often conceptualized as a distal vulnerability factor, our findings support its relevance as a proximal enabler of imminent crises. By reducing fear of death and increasing pain tolerance, capability facilitates the rapid translation of suicidal intent into action when acute distress is present. Empirical evidence indicates that capability differentiates ideators from attempters and interacts with acute stressors to heighten near-term self-aggressive behavior, and that facets such as pain tolerance may fluctuate over short time frames [e.g^48,49^.. In this sense, capability should be understood not only as a background diathesis but also as a dynamic factor that, when activated, can directly increase imminent risk.

Furthermore, *cognitive rigidity* and *impulsivity*, both associated with SCS^6^, have been found to play significant roles in predicting IMSR cases. *Cognitive rigidity*, the inability to adopt alternative thinking patterns, may contribute to a sense of entrapment and hopelessness, making it a pivotal risk factor^7^. This finding aligns with prior findings that suicide attempters exhibit lower cognitive flexibility than suicide ideators^50,51^. Notably, *cognitive rigidity* remained stable predictor throughout the crisis chats, suggesting that it reflects an enduring cognitive trait rather than a transient emotional response. Interestingly, a recent meta-analysis found no association between *cognitive flexibility*, the opposite of rigidity, and suicidal ideation and behavior^50^. However, it is important to note that the authors cited poor ecological validity and inadequate measures of cognitive flexibility in many reviewed studies.

On the other hand, cognitive rigidity may be related to imminent suicide risk only in subgroups such as those diagnosed with autistic spectrum disorder, characterized by cognitive rigidity^52^. Considering that *deliberate self-harm* was also identified as a predictor of IMSR chats, it is reasonable to propose that the most direct way to enhance the capacity for suicidal behavior by overcoming the natural fear of pain is through *deliberate self-harm*^53^. *Deliberate self-harm* predicted significantly IMSR chats approximately halfway through the chat till its conclusion. Thus, desensitization to pain, which lowers the barrier to enact, is not revealed immediately; however, once expressed, it should be noted to clinicians.

The last factor to predict IMSR is *entrapment*, which is part of affective disturbance in the SCS. *Entrapment* represents the desire to escape an unbearable situation, with the perception that all escape routes are blocked^54^. Our findings show that *entrapment* predicts IMSR through most of the time points of the chat, only dropping off at the conclusion. This finding aligns with Rasmussen et al.^55^, who showed that external *entrapment*, such as being caught up in a bad marriage, is implicated in predicting the likelihood of a future suicide attempt. Our findings reveal that the association is not limited to external entrapment but rather to entrapment in general. O’Connor et al.^56^ found entrapment to predict suicide attempts in a clinical sample of patients hospitalized after a suicide attempt, whereas our sample broadens this association to the general population. Importantly, although entrapment and cognitive rigidity are conceptually related, they were operationalized as distinct constructs in our lexicon: entrapment reflects an affective–motivational state (e.g., “I can’t get out of this”), whereas cognitive rigidity refers to a cognitive style characterized by difficulty shifting perspectives or generating alternatives. Prior literature suggests that rigidity may exacerbate or sustain feelings of entrapment during suicidal crises^7,57^. This distinction is reflected in our findings: entrapment emerged as a predictor of IMSR throughout most of the chat, while cognitive rigidity became more prominent in the middle-to-late stages, suggesting that rigidity may act as a cognitive pathway that amplifies or prolongs entrapment over the course of the crisis.

This finding has direct clinical implications: crisis volunteers, who frequently manage several simultaneous chats, must maintain continuous vigilance, as indicators of imminent risk may surface at any stage rather than solely at the outset. Importantly, the temporal perspective also helps to distinguish distal vulnerabilities (e.g., depressive symptoms) from proximal triggers of acute crises, reinforcing the need for dynamic, ongoing assessment rather than static risk categorization.

Consistent with this distinction, certain well-established suicide risk factors—such as *perceived burdensomeness, depressive symptoms*, and *emotional pain*—were negatively associated with IMSR. Closer inspection suggests that these factors may be more characteristic of GSR chats, where individuals often describe ongoing suffering or chronic vulnerability without imminent intent (e.g., ‘Sometimes I feel life is not worth living’). In contrast, IMSR chats were marked by urgent disclosures reflecting immediate risk (e.g., ‘I just took 10 pills’). This pattern indicates that burdensomeness, depression, and emotional pain function more as distal markers of chronic suicidality, whereas acute crises requiring intervention are better predicted by proximal indicators such as explicit planning and intent. This finding is consistent with the broader suicide prevention literature, which differentiates between long-term vulnerabilities (e.g., persistent depression, social withdrawal) and proximal risk factors that act as triggers for acute suicidal crises^7,58^. The distinction between these two categories is clinically significant, as it underscores the necessity of tailoring risk assessment tools to capture time-sensitive indicators of imminent suicide risk rather than relying solely on markers of general suicidal ideation.

This study’s contributions notwithstanding, several limitations should be considered. The predictive accuracy of the model was moderate (AUC = 68.8%), suggesting room for improvement. Notably, this level of performance is broadly consistent with prior literature on suicide risk prediction, where models typically achieve only modest accuracy^17,23^. These parallels highlight both the inherent challenges of forecasting suicidal behavior and the incremental value of our theory-driven, lexicon-based approach. Future research could explore strategies to enhance predictive performance, including integrating multimodal data sources (e.g., speech patterns, behavioral indicators), employing deep learning architectures to capture complex temporal dynamics, and refining lexicon categories through iterative validation. In addition, incorporating longitudinal or repeated-contact data may help capture within-person changes that are particularly relevant to imminent suicide risk. Another key limitation is that the study relied on linguistic representations of psychological variables rather than the direct measurement of the variables. This may indicate a gap between the language patterns identified in the chat transcripts and individuals’ actual psychological states. While language serves as a powerful proxy for cognitive and emotional states^29,30^, it does not always capture the full complexity of suicidal crises. We attempted to address this by conducting validation procedures, including expert coding with substantial inter-rater reliability and phrase representativeness ratings, but some degree of subjectivity is inherent to lexicon-based approaches. Future research should therefore aim to validate these linguistic markers against additional psychological and behavioral data. Future research should aim to validate these linguistic markers using additional psychological and behavioral data. In addition, some predictors may be partially confounded by hotline procedures. For example, disclosures of active suicidal ideation with a specific plan and intent often prompt escalation to the on-call clinical supervisor and, when imminent harm is suspected, referral to police. This structural factor may inflate the predictive strength of such variables beyond their independent psychological contribution. Nonetheless, the fact that suicidal ideation without plan was also associated with IMSR suggests that the model captured additional risk markers beyond protocol-driven triggers. Finally, our model was trained on a specific population—users of an online crisis chat service—which may limit its generalizability. Individuals seeking support through online text-based services may differ significantly from those who do not. Future studies should validate IMSR prediction models across diverse settings and demographic groups to ensure their applicability beyond online crisis intervention platforms.

## Conclusions and practical implications

This study reinforces the need for an integrated approach to IMSR assessment, combining multiple theoretical perspectives such as the Columbia framework, the interpersonal theory of suicide, and the suicide crisis syndrome. By identifying both explicit and implicit risk factors, this study highlights the need to move beyond traditional assessment tools and adopt real-time, dynamic screening methods. The findings also stress the clinical importance of cognitive, affective, and behavioral markers in suicide risk detection. Future advancements in suicide prevention will likely depend on the synergy between clinical expertise and machine learning technologies, creating a more precise and responsive system for identifying individuals in acute distress.

The study underscores the transformative potential of machine learning-based risk assessment in crisis intervention settings. By leveraging natural language processing (NLP), ML models can identify linguistic and psychological markers of IMSR, even when explicit suicidal intent is not disclosed. Given that only 45.9% of individuals at risk disclose their suicidal thoughts^59^, these models can provide a much-needed safety net, ensuring that high-risk individuals receive timely intervention.

From a practical standpoint, crisis hotline services could benefit from real-time AI-assisted monitoring tools that analyze ongoing chat interactions and flag high-risk cases based on dynamic risk patterns. These systems could supplement human decision-making by providing predictive alerts to crisis counselors, enabling more targeted and immediate interventions^29^. Moreover, given that factors such as loneliness tend to emerge later in conversations, as found in this study, AI models could continuously reassess risk levels throughout the chat session rather than relying on static assessments.

Our temporal analysis further underscores the need for risk monitoring throughout the entire chat. Because crisis volunteers often manage multiple chats simultaneously, it is critical to remain alert not only to early disclosures of suicidal intent but also to cues that may emerge later, such as loneliness or self-harm. This highlights the importance of dynamic, continuous assessment in both human practice and AI-assisted systems, ensuring that risk detection does not rely solely on information provided at the start of the conversation.

Additionally, the findings highlight the importance of training crisis hotline volunteers and mental health professionals to recognize subtle linguistic cues associated with IMSR. For example, increased pain tolerance, impulsivity, and cognitive rigidity may indicate a greater likelihood of imminent risk, even in the absence of direct suicide threats. Training programs should integrate insights from ML-based research to enhance early detection capabilities.

## Supplementary Information

Below is the link to the electronic supplementary material.

Supplementary Material 1

Supplementary Material 2

## Data Availability

The datasets generated and analyzed during the current study are not publicly available due to confidentiality agreements with the crisis hotline service but are available from the corresponding author on reasonable request. Requests for access to the data should be directed via email to the corresponding author, Professor Yossi Levi-Belz, at: Josef.Levi@gmail.com.
