# Supplementary material for: Predicting imminent suicide risk in a crisis hotline chat using machine learning
Source: Sci Rep. 2025 Dec 29;15:44742. doi: 10.1038/s41598-025-28704-0 (PMC12748772; doi:10.1038/s41598-025-28704-0)
Supplement: Supplementary file 1 — Supplementary Material 1 [file 41598_2025_28704_MOESM1_ESM.docx]

# Appendix 1:

# Language representations of theory-based constructs and empirically supported psychosocial risk factors for imminent suicide risk (IMSR)

| Construct | Representative Phrases |
| --- | --- |
| Acute anhedonia | I don’t enjoy being with anyone anymore  I lost interest in my job  I don’t find any pleasure in my hobbies  I feel bored of everything |
| Active Suicidal Ideation with Specific Plan and Intent | I found a way to end my life  I already planned how to kill myself  I’m on my way to the bridge to jump  I took lots of pain killers |
| Agitation | I have strange sensations in my body  I feel I wanted to crawl out of my skin  I feel restless, I can’t sit still  I feel a lot of emotional turmoil in my gut |
| Cognitive Rigidity | I cannot change my mind once I come to a conclusion  There is only one solution to my problems  There are no alternatives to where I’m at.  I can’t see a way out |
| Emotional Pain | I feel such inner pain that cannot be stopped  The inner pain is too much to bear  I feel an agonizing emotional pain  This is worse than any physical pain I ever had |
| Entrapment | I feel there is no exit  I feel things will never change  There is no escape from my thoughts  I feel like everything is closing on me |
| Extreme Anxiety | I’m having panic attacks  I feel the blood rushing through my veins  My heart is racing  My chest is so tight |
| Fearlessness of Death | Death doesn’t scare me  I don’t feel uncomfortable when people talk about death  I’m not really upset by the idea that death is the end  I’ve made peace with the idea of death |
| Global Insomnia | I wake up from sleep tired and not refreshed  I had trouble falling asleep  I barely slept  I wake up at nights |
| Hypervigilance | I’m constantly watching for signs of trouble  I feel very intense  I’m always waiting for something bad to happen  I’m on edge all the time, expecting something bad to happen |
| Intense or Persistent Rumination | My thoughts are racing  I’m bothered by thoughts who don’t make sense  I keep thinking about my mistakes  I’m going through it again and again |
| Irritability | I get into a lot of arguments lately  I have temper outbursts I cannot control  I keep bursting at my parents  I snap at people for no reason |
| Pain Tolerance | I can tolerate a lot of pain  The pain involved in dying doesn’t scare me  I love pain  Pain feels normal to me now |
| Rapid Spikes of Negative Emotions/Extreme Mood Swings | I feel deep negative feelings towards myself  I feel like on a rollercoaster  It’s like a storm inside me  I never know what mood I’ll be in |
| Repeated Unsuccessful Attempts to Suppress Negative or Disturbing Thoughts | I'm too weak to stop the disturbing thoughts  I don't have the strength to stop these thoughts from coming in.  The thoughts won't stop  I hit my head against the wall just to make my thoughts stop |
| Ruminative Flood/Cognitive Overload | I feel such pressure in my head from thinking too much  Ideas keep turning over and over in my mind and would not go away  Too many thoughts  I have a headache from too many thoughts |
| Social Withdrawal | I feel isolated from others  I can’t open up to my friends  I push away my family  I evade communication with others |

# Language representations of theory-based constructs and empirically supported psychosocial risk factors for general suicide risk (GSR)

| Construct | Representative Phrases |
| --- | --- |
| Absenteeism. | I barely went to school  I've been calling in sick a lot lately.  ive been in a funk  I’ve been absent a lot this semester |
| Adverse life events | I lost my partner  after losing my job  I’m drowning in debt  I had an ugly divorce |
| Bullying | The kids in class bully me  They call me names  Someone shared private pictures of me  Rumors about me won’t stop |
| Deliberate self harm | I cut myself  I want to hurt myself  I cut my wrists  I burnt myself |
| Depressive symptoms | I am always sad  I’m feeling empty  I cant get out of bed  I’m not eating much lately |
| Drugs and Alcohol | I get drunk a lot  I’m always wasted  I smoke weed everyday  Getting my fix |
| Family suicide history | My friend attempted suicide  My dad killed himself  My partner jumped off a bridge  My sister called herself |
| Hopelessness | I don’t see a future to myself  What’s the point  I have nothing to look forward to  Nothing will ever change |
| Immigration | I wasn’t born here  My parents barely speak the language  I came here when I was  Ever since we moved, I feel |
| Impulsivity | I just do things without thinking  Once I get an idea in my head, I just do it  I don’t plan a head, I go with the flow  I act before I think |
| LGBT | I feel like I can never come out to my family  I’m scared people will find out I’m trans  I’m bi, no one accepts me for who I am  I feel like I'm hiding a part of myself. |
| Loneliness | I don’t have any friends  I have no one to turn to  I’m lonely  I have no one to talk to |
| Past suicidal history | I tried to commit suicide in the past  I took pills when I was  I tried to kill myself  I wanted to jump but my friend stopped me |
| Perceived burdensomeness | the people in my life would be better off if I were gone  I think I make things worse for the people in my life  I feel like a burden on the people in my life |
| Perfectionism | I feel like I’m never good enough  Everyone expects me to be perfect  I'm so afraid of failing  I feel so much pressure to be perfect |
| Psychopathology | I was in a psych ward  my shrink said  I’m on meds  I have mental problems |
| Sexual harassment | I was harassed  I was raped  He forced himself on me  I was sexually abused |
| Suicidal ideation | I wish I was dead  I wish I could go to sleep and never wake up  I wish I was bit alive anymore  I had thoughts about killing myself |
| Thwarted belongingness | I feel disconnected from other people  I feel like an outsider  I feel unwelcome in most social situations  I feel like I don’t belong anywhere |
